# In Pursuit of Stability Enhancement of a Prostate Cancer Targeting Antibody Derived from a Transgenic Animal Platform

**DOI:** 10.1038/s41598-020-66636-z

**Published:** 2020-06-16

**Authors:** Sathya Venkataramani, Robin Ernst, Mehabaw Getahun Derebe, Robert Wright, Jessica Kopenhaver, Steven A. Jacobs, Sanjaya Singh, Rajkumar Ganesan

**Affiliations:** Janssen Biotherapeutics, 1400 McKean Road, Spring House, PA 19477 United States

**Keywords:** Antibody therapy, Thermodynamics

## Abstract

Accelerated timelines necessitate the discovery of fully human antibodies as biotherapeutics using transgenic animals with a notion that such mAbs bypass humanization. A transgenic animal derived mAb (PCa75) targeted against a prostate cancer antigen had several ‘unusual residues’ (rare somatic hypermutations, rSHM, with positional frequency of <1%) that resulted in compromised biophysical properties (Tm = 61 °C and intrinsic stability ΔGu = 24.3 kJ/mol) and a sub-optimal immunogenicity profile. In our quest for quality medicine, we pursued antibody engineering strategies to enhance the stability of PCa75. PCa62, an engineered variant of PCa75, retained function while significantly improving the drug-like attributes of the molecule (Tm = 75 °C and intrinsic stability ΔGu = 63.5 kJ/mol). rSHM is rather prevalent, 18 out the 21 approved transgenic animal-derived antibodies have at least one ‘unusual residue’. Thus, engineering of rSHM remains critical to enhance the stability and minimize immunogenicity risk of biotherapeutics.

## Introduction

Monoclonal antibodies (mAbs) have fundamentally transformed the treatment of complex diseases like autoimmune disorders, cancer, and others over the last two decades^1^. The complex physicochemical makeup of these biologics compared to traditional small molecule drugs is manifested in development challenges associated with immunogenicity, aggregation, chemical stability, and physical stability during drug production and delivery. These challenges are outweighed by the unmatched specificity, potency, and safety of these molecules such that mAbs remain a growing source of therapeutic molecules, particularly in Oncology applications, with over 100 new mAbs entering clinical trials yearly^2^. With over 570 mAbs currently being tested in clinical trials with overlapping targets, mechanisms, and disease indications, optimization of a drug’s physical attributes can result in a significant competitive advantage while also providing better value to patients. Humanized antibodies represent ~43% (i.e. 38 mAbs) of the 89 FDA approved antibodies^2^. Although once tedious and time consuming, synthetic biology breakthroughs, sequencing technologies, and world-wide services for high-throughput antibody engineering have drastically reduced the time required for humanization. More recently several transgenic animal platforms have been developed to discover fully human therapeutic antibodies in rodents, circumventing the iterative process of humanization^3,4^. Indeed, 21 biotherapeutics derived from four different transgenic animal platforms such as UltimAb, Xenomouse, VelocImmune and TransChromo platforms have been approved and marketed while antibodies from other transgenic animals’ platforms like Merus, Harbour Biomed, Ablexis, Wuxi Pharma, Open Monoclonal Technology among others are currently in clinical trials^5^.

Recreating the human immune system in transgenic animals is challenging as antibody genes are assembled by V-D-J recombination and further diversified by somatic hyper-mutations to enhance specificity. The somatic hyper-mutation (SHM) process may result in non-human germline mutations that are selected to be compensatory for deleterious effects on stability during maturation *in vivo*. Some of these SHM tends to be low frequency (<1%) unusual rare SHM (rSHM). These mutations present a challenge for humanization, as the compensatory effects may not be translated to the humanized molecule, ultimately resulting in a destabilized antibody. This phenomenon is also mimicked in antibody engineering, where it has been consistently observed that *in vitro* affinity matured antibodies are less thermostable than the parental antibody from which they are derived and further selections are required for improving the stability of the antibodies that mimics the natural process of somatic hypermutation *in vivo*^6^. Conformational stability is commonly used as surrogate measurement for successful antibody stabilization as thermal stability is correlated with high expression, easier purification, longer shelf life^7^ and optimal pharmacokinetic/pharmacodynamic properties^8^. A recent study suggests that the thermal stability of Fab portion of the antibody is key for overall molecule stabilization^9^.

In this work, we describe the results of multiple first principle-based methods used to determine the intrinsic biophysical properties of a transgenic rodent derived mAb generated against a prostate target protein. PCa75 was found to have framework mutations caused by SHM that compromised the conformational stability of the molecule, leading to a higher propensity for aggregation. Structural modeling and sequence alignment were used to re-engineer the selected set of framework positions of this antibody to produce a new candidate that retains high affinity target binding with increased conformational stability, reduced immunogenicity risks and hence desirable manufacturable properties.

## Results

### Discovery, Engineering and Structural Assessment of PCa75

The extracellular domain of human prostate cancer antigen was produced recombinantly and used to immunize transgenic rodents for therapeutic antibody discovery. From the initial hybridoma hits, PCa75 was shown to have all desirable functional attributes including a binding affinity of 247 pM to soluble recombinant antigen (Table 1). A subsequent thermal stability assessment measured with differential scanning fluorimetry (DSF) determined relatively low conformational and colloidal stabilities with values of the onset of unfolding of 52.9 °C, Fab unfolding of 59.0 °C and onset of aggregation of 61.5 °C (Table 2).

**Table 1:** Protein binding affinity by SPR. Parameters from Affinity measurement done by SPR are provided in this table. Association constant *k*_a_ (M^−1^ s^−1^), dissociation constant *k*_d_ (s^−1^) and equilibrium constant K_D_ (M) are included.

mAb	*k*_a_ (M^−1^ s^−1^)	*k*_d_ (s^−1^)	*K*_D_ (pM)
PCa75	6.70 × 10^5^	1.66 × 10^−4^	247.0
PCa62	5.41 × 10^5^	1.74 × 10^−4^	323.3

**Table 2:** Thermal stability and Intrinsic Stability parameters (DSC and DSF). Thermal Stability Parameters determined from DSC (onset of unfolding and Fab domain unfolding Tm) and Intrinsic Stability Parameters from ICD experiments. ΔG_u_1, ΔG_u_2, c50 are the calculated parameters from 2-state and 3-state fitting of GdnCl induced denaturation curves generated in nano DSF experiment.

mAb	Onset (DSC) (°C)	Tm (Fab): DSC (°C)	ΔG_u_1 (kJ/mol)	c50 [M]	ΔG_u_2 (kJ/mol)	c50 [M]
PCa75	52.9	61.8	24.3	1.8	NA	NA
PCa62	59.3	75.5	63.5	1.9	37.2	2.9

**Figure 1:**
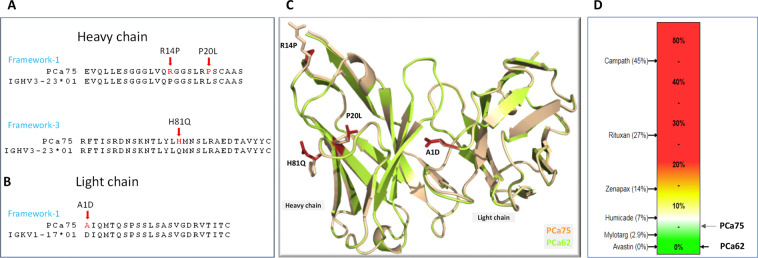
(**A**) Sequence alignment of the framework-1 and framework-3 region of PCa75 and human germline heavy chain. The somatic hypermutations are indicated by arrows. (**B**) Sequence alignment of the framework-1 region of PCa75 and human germline light chain. (**C**) Molecular model of Fab fragment of PCa75 (wheat) and PCa62 (green) overlaid and the SHM residues are highlighted as stick model. (**D**) EpiVax *in silico* immunogenicity assessment shows PCa62 has reduced risk compared to PCa75. In general, proteins having EpiMatrix score >20 tend to be more immunogenic, while proteins with score < −20 tend to be immunologically inert. A select set of mAbs with observed % ADA (Anti-Drug Antibody) incidence are indicated.

**Figure 2:**
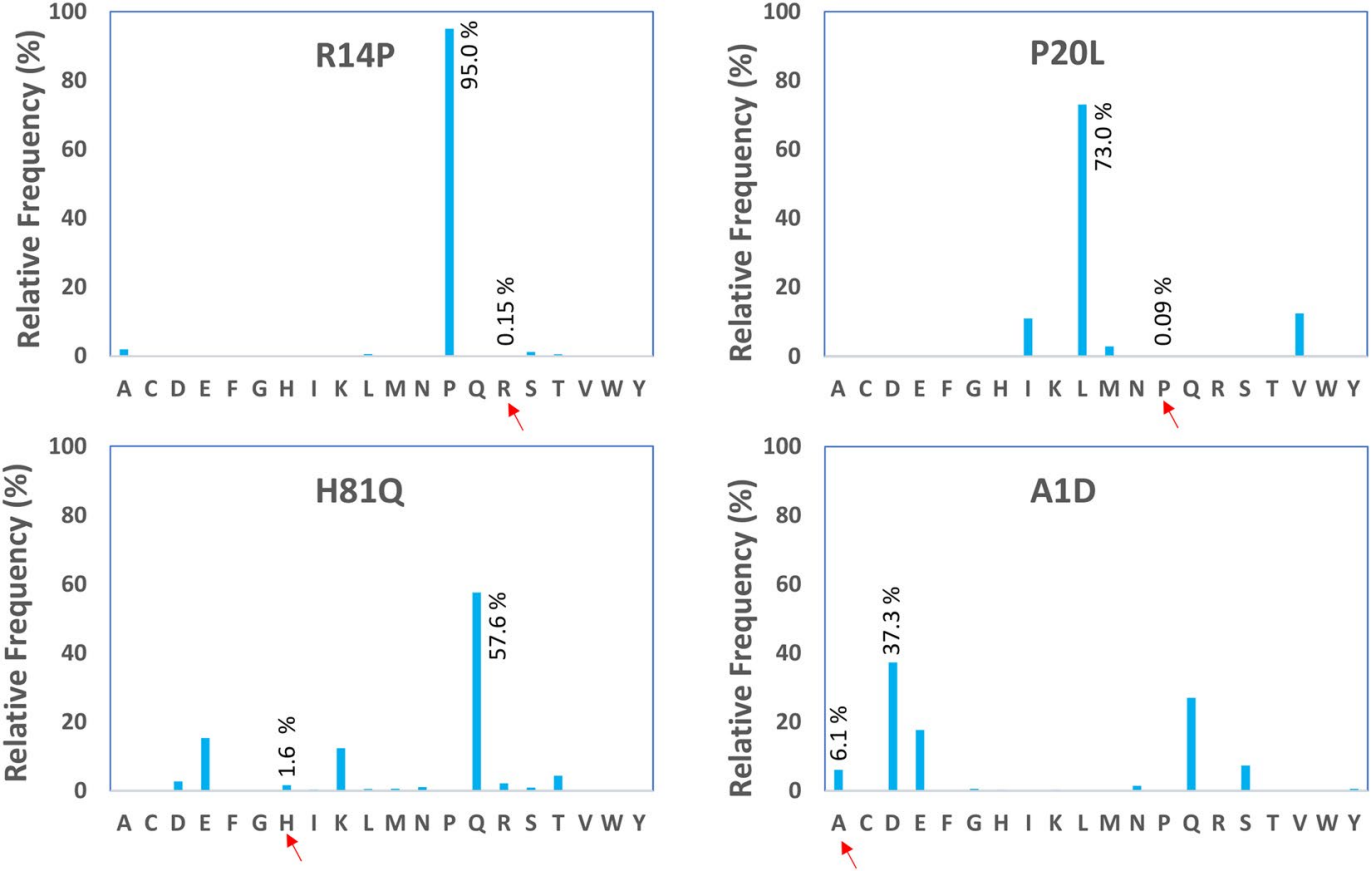
Relative positional frequency of the four SHM observed in PCa75 and PCa62 using the abYsis portal.

A closer investigation and sequence alignment of light and heavy chains of PCa75 shows four somatic hypermutations (positions 14, 20, and 81 of the heavy chain and positions 1 of the light chain) different from its germline precursors (IGKV1-17*01 and IGHV3-23*01, Fig. 1A,B). The SHM Arginine found at position 14 is rare (frequency 0.15%) compared to the most frequently found Proline residue at this position (Proline residue frequency 95.03%). The frequency of the SHM, Proline, found at position 20 of the heavy chain is also low (frequency 0.09%) compared to most frequently found Leucine residue (frequency 73.02%). The frequency of finding the third SHM, Histidine at position 81 is relatively rare too (frequency 1.60%) compared to the most frequently found Glutamine residue typically found at that position (frequency 57.58%). Hydrogen-Deuterium Exchange Mass Spectrometry (HDX-MS) was used to define the Antigen binding paratope of PCa75. None of the four framework mutations fell within the binding paratope, leading to the hypothesis that mutations of these positions could be used to optimize conformational stability while maintaining target binding. In the heavy chain, EpiVax assessment indicated that Pro20 and His81 were part of immunogenic 9-mer Epibar (LR**P**SCAASG and YL**H**MNSLRA). A series of 10 mutant forms of PCa75 were produced to systematically test the effects of each framework mutation on binding and stability. A molecular model for the parental Fab (PCa75) and a representative variant (PCa62) was constructed (Fig. 1C), with minimal changes in r.m.s.d. between the two models observed. Replacement of Leu20 (HC) from the germline sequence with proline in PCa75 may reduce hydrophobic packing with HC-V123, Y101 and W40. Additionally, the Q81H mutation found in PCa75 has the potential to reduce several polar contacts with S17 and N91. Using the molecular model, along with an assessment of unusual residues (Fig. 2) using abYsis^10^ software-based, a sequence alignment was used to design amino acid substitutions at multiple positions to improve stability. The immunogenicity potential of each mutation was also calculated using EpiVax (Fig. 1D). In general, proteins having EpiMatrix score >20 tend to be more immunogenic, while proteins with score < −20 tend to be immunologically inert^11^. Each mutant was expressed in CHO cells, purified and binding to the target protein were determined. In addition, the conformational stability was measured by differential scanning fluorimetry monitoring intrinsic tryptophan fluorescence. The summary of each attribute for each mutant is detailed in Table 3. All engineered variants have a 5–9 °C increase of Fab Tm and an increase in onset of aggregation of 6–14 °C compared to the parent PCa75 antibody. All the three mutations (R14P, P20L and H81Q) on the heavy chain had profound effect on the overall immunogenicity risk reduction. These replacements led to the formation of several 9-mer Tregitopes (VQ**P**GGSLRL, LR**L**SCAASG, LYL**Q**MNSLR, YL**Q**MNSLRA, L**Q**MNSLRAE). Cumulative consideration of immunogenicity potential and significant improvements in conformational and colloidal stability led to the selection of PCa62 as the most promising candidate.

**Table 3:** EpiVax scores (Heavy and Light Chain), Fab Tm (DSC), mAb Tagg (DSF) values of all 10 variants including PCa75.

Variants	HC mutations			LC mutation	EpiVax Score (Heavy Chain)	EpiVax Score (Light Chain)	Fab (Tm) °C	mAb (Tagg) °C	*K*_*D*_ (SPR, pM)
	14	20	81	1					
**PCa75**	**R**	**P**	**H**	**A**	**1.99**	**−19.3**	**61.8**	**61.5**	247.0
PCa46	P	L	Q	A	−44.3	−19.3	68.3	70.5	279.2
PCa47	P	L	H	A	−8.9	−19.3	68.1	70.2	284.5
PCa50	R	L	Q	A	−29.6	−19.3	66.2	67.8	281.6
PCa51	R	L	H	A	5.75	−19.3	65.9	68	298.6
PCa60	R	L	Q	A	−29.6	−33.6	69.7	72.3	323.9
**PCa62**	**P**	**L**	**Q**	**D**	**−44.3**	**−33.6**	**69.4**	**75.7**	323.3
PCa63	P	L	H	D	−8.93	−33.6	69.6	75.6	325.3
PCa64	P	P	Q	D	−37.3	−33.6	68.2	70	372.3
PCa65	P	P	H	D	−2.02	−33.6	68.5	70	405.8
PCa66	R	L	Q	D	−29.6	−33.6	69.4	72.2	405.4

### Comparison of biophysical properties of PCa62 and PCa75

A number of biophysical measurements were conducted to understand the effects of the germline reversions contained in PCa62 on the overall biophysical properties of this molecule compared to the parent. PCa75 and PCa62 existed as >97% monomer after 2-step purification as measured with sedimentation velocity analytical ultracentrifugation (SV-AUC) (Fig. 4B), demonstrating the production of good quality starting material for the additional biophysical characterization.

**Figure 3:**
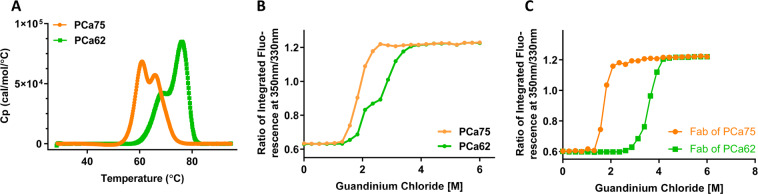
Intrinsic Properties Characterization of PCa75 and antigen. (**A**) Differential Scanning Calorimetry melting profile of PCa75 (orange) and PCa62 (green). Heat capacity Cp (kcal/mol/°C) is plotted against the temperature (°C) (**B**) Isothermal Chemical Denaturation in GdnCl as monitored by change in fluorescence intensity ratio 350/330 nM for PCa75 (orange) and D) PCa62 (green). (**C**) Isothermal Chemical Denaturation in GdnCl as monitored by change in fluorescence intensity ratio 350/330 nM for the Fab of PCa75 (orange) and the Fab of PCa62 (green).

Table 1 demonstrates that PCa62 retains high affinity binding (323.3 pM) to the target antigen, confirming that binding activity was retained following engineering. Being a high-throughput assay, differential scanning fluorimetry (DSF), which is a fluorescence-based assessment for thermal stability may lack precision and accuracy. Hence, differential scanning calorimetry (DSC) was also used to confirm the increase in conformational stability for PCa62 determined with differential scanning fluorimetry (DSF) (Table 3). PCa75 and PCa62 melt with 2 transitions, typical of IgG4 antibodies (Fig. 3A and Table 2). The DSC transition at 65 °C was interpreted to result from concomitant melting of the CH_2_ and CH_3_ domains while the transitions at 61.8 °C and 75.7 °C were assigned to the Fab domains of PCa75 and PCa62 respectively, demonstrating a 13.9 °C increase in Fab in melting temperature for the engineered variant. Isothermal Chemical Denaturation (ICD) at a single temperature is another way to measure antibody stability that can provide complementary information to thermal melting^12^. In an ICD experiment, mAb at a single concentration is incubated at increasing concentrations of a chemical denaturant for a minimum of 12–16 hours before measuring its conformational change. The change in F350/F330 fluorescence ratio is used to determine the fraction unfolded at each measured denaturant concentration. The Gibbs free energy of unfolding (ΔG_u_) calculated from the fitting curves is an indicator of intrinsic conformational stability of the mAb at a particular temperature^13^. Another important parameter from this fitting is c50, which represents the concentration of the denaturant at which 50% of the antibody is unfolded.

Figure 3B provides the ICD unfolding curves of PCa75 and PCa62 measured at 25 °C in Guanidine-Hydrochloride (GdnCl). PCa75 exhibits a single transition with ΔG_u_ of 24.3 kJ/mol while PCa62 shows 2 unfolding transitions, with the first transition ΔG_u_ of 63.5 kJ/mol and a second transition of ΔG_u_ of 37.3 kJ/mol. The midpoint of transition however is the same for both mAbs (c50 = 1.9 M GdnCl). The approximate 2.5-fold increase in the free energy of unfolding of the first transition of PCa62 clearly demonstrates that this mAb is intrinsically more stable than PCa75 due to germline optimized Fab domain, in agreement with the thermal denaturation results. In addition, we have generated just the Fab domains alone of both PCa75 and PCa62. Chemical denaturation curves were generated for the Fab domains (Fig. 3C) under the same experimental conditions adopted for the mAbs. The midpoint of transition for the PCa75 Fab is 1.7 M while for the PCa62 Fab is 3.6 M GdnCl. Also, the free energy of unfolding of PCa62 Fab is 42.5 kJ/mol while that of the Fab of PCa75 is 34.8 kJ/mol. The conformational stability of both Fab molecules was also assessed by DSF and the data confirmed the enhanced thermal stability of PCa62 (Tm of PCa62 Fab = 81.5 °C; Tm of PCa75 Fab = 61.8 °C).

**Figure 4:**
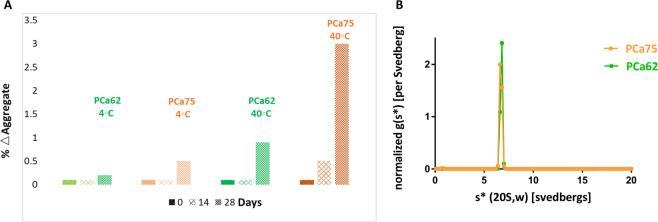
(**A**) Storage (4 °C) and Accelerated (40 °C) stability of PCa62 and PCa75 monitored for a month. Change in aggregate level between time zero and 1 month is plotted against the days **(B)** Analytical Ultracentrifugation Sedimentation Velocity (AUC-SV) runs of both PCa62 (orange) and PCa75 (blue). The normalized g(s*) distribution for both PCa75 (orange) and PCa62 (green) sedimentation velocity runs are shown. Global fitting analysis was done by SEDANAL v697 and the data was fitted globally to two species, non-interacting model. Both purified antibodies exhibited >97% monomer as analyzed.

Storage and accelerated degradation were studied for PCa75 and PCa62 in order to determine if increase in Fab stability for the engineered variant may result in better pharmaceutical properties. Each molecule was stored for 28 days in PBS at 4 °C and 40 °C and degradation monitored with analytical size exclusion chromatography at time points 0 days, 14 days, and 28 days. Figure 4A plots the changes in aggregate levels at each time point for both mAbs. PCa62 had less than 0.3% aggregates at 4 °C and less than 1% aggregates at 40 °C for a month while PCa75 showed 0.5% and 3% aggregation increase under these same conditions. As consistent with many literatures, higher thermal stability correlates with lower propensity for aggregation^9,14^. Collectively, these biophysical profiling data led to the selection of PCa62 as a therapeutic lead candidate. Extended stability studies and critical quality attributes such as charge and clipped variants, particles etc., would be necessary for further understanding of PCa62’s CMC and manufacturing attributes.

### Binding of PCa62 to recombinant antigen in Human Serum

**Figure 5:**
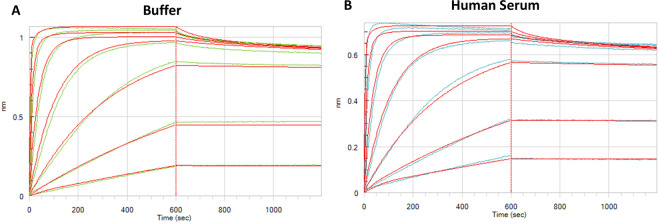
Serum interference data for PCa62 as measured by Octet RED384 in buffer (**A**) and in 50% human serum (**B**). Experimental data shown in green (in buffer) and blue (in human serum) while fitted data is red. Biolayer interferometry was used to determine if the presence of human serum changed the association rate of PCa62 for antigen. No major impact in binding was observed in the presence of 50% serum compared to buffer only.

It is critical to understand the stability of lead candidates in serum since circulation in serum affects both potency and alters biophysical properties of the molecules. In the serum interference assay, an Octet RED384 instrument is used to measure the association rates of lead candidate in buffer and in 50% human serum to the biotinylated soluble target protein bound to streptavidin tip. PCa62 was tested using a 3-fold dilution series of 300 nM down to 0.41 nM concentration against 1.5 μg/mL of biotinylated target protein. The ratio of association rates of binding of antibody to the target in both buffer and serum is calculated to determine if the binding of lead to the target is retained in the serum. Figure 5 shows that the profiles of PCa62 binding to antigen in 50% serum and buffer as measured with biolayer interferometry. Association rates of 2.04 × 10^5^ (M^−1^s^−1^) and 2.05 × 10^5^ (M^−1^ s^−1^) were measured in buffer and serum respectively, indicating a lack of nonspecific interactions with serum proteins.

## Discussion

Antibody engineering methodologies such as humanization and framework shuffling have been the core of discovering biotherapeutics^15–17^. The advent of several transgenic animal platforms enabled the discovery of fully human therapeutic antibodies in rodents, circumventing the process of humanization^3–5^. A significant number of antibody sequences obtained from transgenic animals contain somatic hypermutations in the framework and CDR regions that may result in unusual or low frequency residues that impact the stability and immunogenicity of biotherapeutics. Initial discovery of anti-prostate mAb PCa75 from a transgenic animal platform resulted in a molecule compromised of its intrinsic physical properties, resulting in lower conformational and colloidal stability and thus potentially posing greater challenges for manufacturing and development. A series of variants with germlining mutations in either the heavy chain or light chain, or combination of heavy and light chains, were expressed and assessed for binding, functional activity and biophysical properties. Binding, immunogenicity scores, and thermal stability were at the top of our screening funnel to rapidly select the most promising lead from the library of variants. The EpiMatrix software^11^ was used to predict the immunogenicity and overall immunogenic potential of a biologic. EpiMatrix also identifies individual T cell epitope clusters contributing to its immunogenicity. Both conformational and colloidal stability were included in this early screen as they are amenable to high-throughput screening and are well demonstrated manufacturability parameters that predict stability, shelf life and successful drug development. Simultaneous assessment of both parameters is a very powerful approach for long term stability determination as a subtle change in conformation can eventually lead to aggregation, loss of activity and high immunogenicity risks^18^. PCa62 was selected that has shown the ideal EpiVax Score and enhanced thermal stability while retaining binding.

Measuring inherent stability of an antibody even at lower temperatures is one of the effective ways to understand and select a therapeutically viable candidate that can withstand all stresses during manufacturing, storage and shipment^19^. Undoubtedly thermal denaturation experiments are one of the commonest stability determination tools available as high throughput for rank ordering molecules. However, the existing challenge is to accurately calculate the intrinsic stability at lower temperatures purely based on higher temperature data. This calculation is error-prone because thermal melting is often irreversible due to aggregation which precludes to extrapolate the reliable stability parameters to lower temperatures^19^. Also, temperatures of most interest to predict the stability of the lead candidate are storage (4 °C and 25 °C) and physiological (37 °C). Measuring stability with isothermal chemical denaturation is used to measure ∆Gu as this parameter determines the population of any conformational state (native, partially unfolded, denatured) at the given concentration of denaturant. Tm is a qualitative measurement that can be done initially in a high throughput fashion with less material to exclude candidates that do not meet the necessary threshold for thermal stability while ∆G_u_ is a quantitative measurement that provides more insights into aggregation^20^. There are increasing evidences from research groups to show that differential scanning fluorimetry (DSF) is routinely used for determining conformational and colloidal stability of antibody-based therapeutics. In particular, measuring ΔG_u_ of unfolding is an invaluable tool for selecting appropriate mAb formulations and predicting long term storage stability^12,21,22^. The data reported from DSF is the average of n = 3 replicates that verified the precision and accuracy of the technique (ΔG_u_ is +2 kJ/mol and c50 is +0.2 M). In a recent study for a series of therapeutic antibodies, nanoDSF was able to detect thermal transition signals with high precision at a wide concentration range^23^.

Compared to PCa75 that has ∆Gu of 24.3 kJ/mol, sequence optimized PCa62 has 2.5-fold increase in conformational stability (∆G_u_ = 63.5 kJ/mol) which correlates well with the increased thermal stability observation. This is in line with accelerated stability results that supported the selection of PCa62 which has very low propensity to aggregate (<1%) for a month stored at 40 °C meeting the quality attributes of panel of FDA/EMA approved and clinical stage mAb candidates^24^. While characterizing mAbs early on to screen for stability and aggregation, equally important is to understand the specificity and serum behavior to select the right candidate that has favorable pharmacokinetic properties without off-target binding. Our *in vitro* serum-based assay has clearly demonstrated that PCa62 retains its stability and binding in human serum predicting a successful development profile.

Genetically engineered transgenic animals with manipulated immune systems are expected to produce affinity-matured, fully-human variable regions in antibodies that can easily be fast tracked to development phase skipping antibody engineering steps. However, sequence analysis of the 21 approved mAbs that came from transgenic animal platforms showed that 18 of those have a minimum of 1 unusual residue (Table 4). It remains to be seen whether engineering rSHM on these 18 mAbs would also result in improved biophysical properties. The cost to develop one new drug has been estimated to be ~2.6 billion with only a <12% approval rate for each drug entering clinical studies^25^. Some of this low success rate may be attributed to molecules progressing from early to late stage clinical trials with sub-optimal stability and safety profiles. Comprehensive analysis of biophysical properties of 137 clinical stage mAbs was presented recently with a key observation that approved mAbs had very few red flags with respect to developability criteria^26^. Most recent publication highlighted the necessity of engineering strategies for affinity matured B-cell derived antibodies that impaired the biophysical properties^27^.

**Table 4:** Number of unusual residues in FDA approved antibodies derived from transgenic animals.

International non- proprietary name	Target; Format	unusual residues	
		Heavy chain	Light chain
Panitumumab	EGFR; Human IgG2	1	0
Denosumab	RANK-L; Human IgG2	0	1
Evolocumab	PCSK9; Human IgG2	4	2
**Brodalumab**	**IL-17R; Human IgG2**	**0**	**0**
Erenumab	CGRP receptor; Human IgG2	3	1
Nivolumab	PD1; Human IgG4	3	0
Dupilumab	IL-4R α; Human IgG4	1	2
Ustekinumab	IL-12/23; Human IgG1	2	3
Canakinumab	IL-1β; Human IgG1	2	3
**Golimumab**	**TNF; Human IgG1**	**0**	**0**
Ofatumumab	CD20; Human IgG1	1	0
Ipilimumab	CTLA-4; Human IgG1	1	1
Secukinumab	IL-17a; Human IgG1	1	1
Alirocumab	PCSK9; Human IgG1	2	1
**Daratumumab**	**CD38; Human IgG1**	**0**	**0**
Olaratumab	PDGFRα; Human IgG1	4	0
Bezlotoxumab	Clostridium difficile enterotoxin B; Human IgG1	3	0
Sarilumab	IL-6R; Human IgG1	1	1
Durvalumab	PD-L1; Human IgG1	0	1
Burosumab	FGF23; Human IgG1	1	1
Cemiplimab	PD-1; Human mAb	1	7

In summary, transgenic animal derived lead PCa75 was germlined resulting in an ideal score for both biophysical and immunogenicity profiling of the resulting variant PCa62. A limitation in this report is that it is indeed a single case study. However, we have observed a similar trend in several of our transgenic animal derived mAbs (anti-PSMA and anti-DLL3) with unusual amino acid residues where antibody engineering was a mandate to improve overall drug like profile of lead candidates (Supplementary Figures S1 and S2). While extensive stability studies leading to correlation between critical quality attributes and binding are warranted to guarantee success in manufacturing, critical experiments such as conformational/colloidal stabilities, serum behavior, 40 °C accelerated stress can provide early guidelines for lead selection. Thus, engineering of rare somatic hypermutations occurring during antibody generation in these animals remains an important strategy to enhance the stability and mitigate immunogenicity risk of biotherapeutics.

## Methods

### Antibody Discovery and Germline Optimization

The monoclonal antibody (PCa75) was discovered by immunization with the recombinant human soluble Antigen protein in a transgenic rodent. Following 89-day immunization regimen, lymph nodes from the animal were harvested and used to generate hybridomas and the hybridoma supernatants were screened for binding to recombinant human antigen by ELISA. Based on the screening results, clone PCa75 met the criteria for binding and was further germline optimized to produce clone PCa62. Full length antibodies and Fab domains of PCa75 and its engineered variants were generated using standard methods. Sequence alignment of the variable heavy and light chain regions of PCa75 with human germline sequences for VH and VL using the abYsis portal indicated several somatic hypermutations (SHM) within the framework region^10^. Within the framework region, three somatic hypermutations were observed in VH (R14P, P20L, H81Q) and one SHM (A1D) was observed in Vk. The antibody sequences were analyzed for potential immunogenicity using the T-regulatory (Treg) adjusted scores from the EpiVax Epimatrix *in silico* immunogenicity prediction program^11^. EpiVax program computationally calculates the binding potential to the most common HLA molecules within each of the “supertypes”. The report provides results that are representative of >90% of human populations worldwide without the necessity of testing each haplotype individually. EpiMatrix score is the Z-score of predicted T-cell epitopes contained within a given protein sequence. In general, proteins having EpiMatrix score >+20 tend to be more immunogenic, while proteins with score < −20 tend to be immunologically inert.

### Binding affinity of Antibody variants by Surface Plasmon Resonance

The binding of anti-prostate mAbs to a recombinant human Antigen was measured by BIAcore 8K SPR (GE). The format of the assay was to capture the mAbs using a high density anti-human Fc surface, then injecting the concentration series of analyte, using a single cycle kinetics method. The BIAcore 8 K SPR platform has 2 flow cells, each with 8 spots, flow cell 1 was the reference flow cell and flow cell two was used to capture mAbs. Goat anti-human Fc IgG (Jackson Immuno research, Cat# 109-005-098) was directly immobilized via amine coupling at 30 µg/mL in 10 mM acetate buffer, pH 4.5 on flow cells 1 and 2, on CM5 Sensor Chip (GE) with a flow rate of 30 µL/min in HBSP (GE) buffer. The mAbs were captured on the anti-human Fc IgG surface at 0.5 ug/ml (~200–300 RU) on flow cell 2. The running buffer was then changed to HBSP + 100ug/ml BSA. The human target protein at 30 nM concentration in 3-fold dilution series was injected from low to high concentration using single cycle kinetics method. The off-rate was monitored 30 minutes after the last or highest concentration injection and then the surface was regenerated using 0.8% phosphoric acid (Bio-Rad). A buffer blank run, capturing the same mAbs and using the same conditions of sample run was also completed. The raw data were processed by subtracting two sets of reference data from the response data: 1) reference flow cell 1 subtracted from sample flow cell 2 and 2) buffer blank run from experimental run. The processed data at all concentrations for each mAb were globally fit to a 1:1 simple Langmuir binding model to extract estimates of the kinetic (kon, koff) and affinity (K_D_) constants.

### Differential Scanning Fluorimetry (DSF)

Thermal stability of a sample was determined by NanoDSF method using an automated Prometheus instrument. Measurements were made by loading sample into 24 well capillary from a 384 well sample plate. Duplicate runs were performed for each sample. Prometheus NanoDSF user interface (Melting Scan tab) was used to set up the experimental parameters for the run. The thermal scans for a typical IgG sample span from 20 °C to 95 °C at a rate of 1.0 °C/minute. Dual-UV technology monitors intrinsic tryptophan and tyrosine fluorescence at the emission wavelengths of 330 nm and 350 nm, and this ratio (F350 nm/F330 nm) is plotted against temperature to generate an unfolding curve. Using back reflection technology, the instrument can measure the on-set temperature of aggregation which is plotted as mAU (Attenuation Units) against temperature. Nano DSF is used for measuring thermal unfolding parameters (Tm and Tagg) of both PCa75 and PCa62 at 0.5 mg/mL concentration in Phosphate Buffered Saline, pH 7.4.

Chemical denaturation experiments were carried out by incubating the purified mAbs in different concentrations of Guanidium chloride (GdnCl) starting from 0 M to 6 M overnight at room temperature. Next day, intrinsic fluorescence was measured using NanoDSF at 25 °C and the ratio of F350 nm/F330 nm is plotted at each concentration of GdnCl to generate an unfolding curve that was fitted either by two-state or three-state unfolding equations^13^ to obtain the parameters such as free energy of unfolding (ΔGu); concentration of denaturant at which 50% of molecules exist as unfolded (c50 [M]).

### Differential Scanning Calorimetry (DSC)

The thermal stability of both antibodies was characterized by capillary VP-DSC microcalorimeter (Microcal Inc. Northampton, MA). The concentration of protein was 1.0 mg/mL measured at a scan rate of 1 °C/min with a cell volume of 0.450 mL. Temperature scans were performed from 25 to 100 °C. A buffer reference scan was subtracted from protein scan and the concentration of protein was normalized prior to thermodynamic analysis. The data was plotted in Origin 7.0 (OriginLab, Northampton, MA) and subsequent thermodynamic analysis was carried out on pre- and post-transition baseline corrected data. The DSC curve was fitted using non-two-state model to obtain the enthalpy and apparent transition temperature (T_m_) values.

### Short term stability (4 °C and 40 °C)

Antibodies were concentrated to 10 mg/mL using 30 kDa MWCO Amicon Ultra centrifugal filter devices (Millipore) at room temperature. Concentrated mAbs were confirmed for final concentration by UV280 spectrophotometer and tested by analytical size exclusion chromatography (SEC-HPLC) for monomer percentage. SEC-HPLC experiment was performed on an Agilent 1260 infinity series II HPLC setup and data analyzed in Open Lab Chemstation interface. A TOSOH TSKgel BioAssist G3SWXL 7.8 mm I.D. x30cm, 5um column was used with mobile phase (0.2 M Na-phosphate pH 6.8) at a flow rate of 1 ml/min and detection @280 nm. Both mAbs were then incubated at 4°C and 40°C for 4 weeks. Aliquots were drawn at regular intervals and integrity was checked by SEC-HPLC.

### Analytical Ultra Centrifugation

Samples were loaded into centrifuge cells equipped with 1.2 cm Beckman centerpieces (rated to 50 K rpm) and quartz windows. The cells are assembled and torqued to 130 lbs. The centrifuge cells were placed into an An-50 (8 hole) or An-60 (4 hole) rotor and placed within the Beckman Optima AUC chamber. The temperature of the AUC was equilibrated to 20.5 °C for at least one hour with the rotor in the chamber before initiating the run. Runs were performed at 40 K rpm for mAb sample with scan count (250 scans), frequency of scan collection (90 seconds), data resolution (10 μM). Absorbance data was collected at 280 nm. Initially the data were analyzed using the software program DCDT^28^ in order to determine the meniscus position and to observe the sedimentation distribution profiles. The data were then analyzed using the direct boundary fitting software^29^. The meniscus position was determined by DCDT+, the baseline was set at 7.2, and manually choosing the fit range. A two species, non-interacting model was used to fit the data with the first species corresponding to monomer and the second species corresponding to dimer.

### Binding in Human Serum

For testing the binding of PCa62 to the soluble Antigen in human serum, Biolayer interferometry (BLI) method is used. The assay format is Streptavidin (SA) biosensor equilibration in either blocking buffer (1xPBS, 0.1% BSA, and 0.02% Tween) or 50% human serum in Octet RED384 instrument. The antibody was tested in buffer and serum using a 3-fold dilution series of 300 nM down to 0.41 nM against 2.5ug/mL of biotinylated target protein. The association rates measured in both buffer and serum are compared.

## Supplementary information

Supplementary Information.
