# Supplementary material for: In Pursuit of Stability Enhancement of a Prostate Cancer Targeting Antibody Derived from a Transgenic Animal Platform
Source: Sci Rep. 2020 Jun 16;10:9722. doi: 10.1038/s41598-020-66636-z (PMC7298045; doi:10.1038/s41598-020-66636-z)

# In Pursuit of Stability Enhancement of a Prostate Cancer Targeting Antibody Derived from a Transgenic Animal Platform

*Sathya Venkataramani[1], Robin Ernst[1], Mehabaw Derebe[1], Robert Wright[1], Jessica Kopenhaver[1], Steven Jacobs[1], Sanjaya Singh[1] and Rajkumar Ganesan[1*]*

*[1]Janssen Biotherapeutics, 1400 McKean Road, Spring House, PA 19477*

[*]Correspondence

Rajkumar Ganesan
Janssen Biotherapeutics
1400 McKean Road,
Spring House, PA 19477
Ph: (215) 628-6880
Email: rganesa3@its.jnj.com

## Supplementary information

**Figure S1**: (**a**) Sequence alignment of an anti-PSMA heavy chain (VH) with human germline IGHV4-39*01 and its engineered variant. An unusual framework residue (Ile) at position 68 was re-engineered to Thr. (**b**) Relative positional frequency of the SHM using the abYsis portal. (**c**) Intrinsic properties characterization of anti-PSMA using Differential Scanning Fluorimetry (DSF). An improvement in both Tm and Tagg was observed for the engineered variant.

**Figure S2**: (**a**) Sequence alignment of an anti-DLL3 heavy chain (VH) with human germline IGHV3-13*05 and the light chain (Vk) with human germline IGKV1-5*03 and a series of engineered variants. Two unusual framework residues at position 85 (in the VH) and at position 84 (in VL) were re-engineered. (**b**) The composition of mutations in VH and Vk for the three engineered variants (**c**) Intrinsic properties characterization of an anti-DLL3 using Differential Scanning Fluorimetry (DSF). An improvement in both Tm and Tagg was observed for the engineered variant. (**d**) Relative positional frequency of His85 (in VH) using the abYsis portal. (**e**) Relative positional frequency of Glu84 (in Vk) using the abYsis portal.

## Supplementary Figure – S1

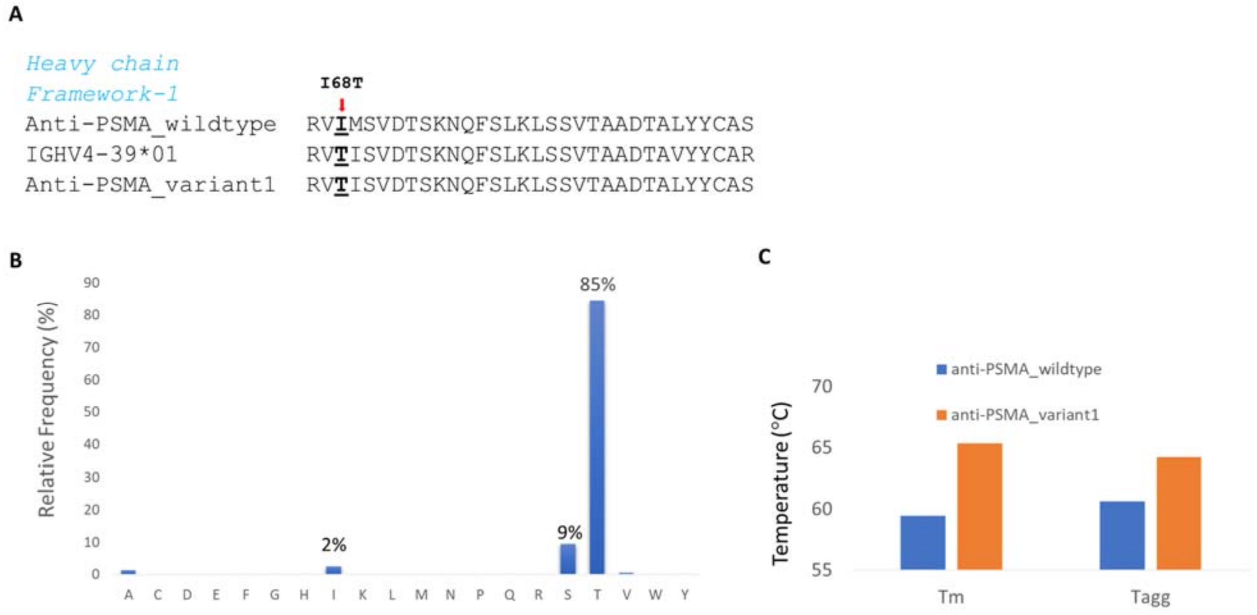

# Supplementary Figure – S2

**A**

**Heavy chain_Framework-3**

```
ANTI-DLL3_HC                 RFTISRENVKHSLYLQMNSLRVGDTAVYFCAR
IGHV3-13*05                  RFTISRENAKNSLYLQMNSLRAGDTAVYYCAR
ANTI-DLL3_variant-1_HC       RFTISRENVKNSLYLQMNSLRVGDTAVYFCAR
ANTI-DLL3_variant-2_HC       RFTISRENVKNSLYLQMNSLRVGDTAVYFCAR
ANTI-DLL3_variant-3_HC       RFTISRENVKHSLYLQMNSLRVGDTAVYFCAR
```

**Light chain_Framework-3**

```
ANTI-DLL3_LC                 GVPSRFSGSGSETEFTLTISSLQPDDFATYYC
IGKV1-5*03                   GVPSRFSGSGSGTEFTLTISSLQPDDFATYYC
ANTI-DLL3_variant-1-LC       GVPSRFSGSGSETEFTLTISSLQPDDFATYYC
ANTI-DLL3_variant-2-LC       GVPSRFSGSGSGTEFTLTISSLQPDDFATYYC
ANTI-DLL3_variant-3-LC       GVPSRFSGSGSGTEFTLTISSLQPDDFATYYC
```

**B**

| # | Name | Heavy chain | Light chain |
|---|---|---|---|
| 1 | Anti-DLL3-wildtype | DLL3_HC | DLL3_LC |
| 2 | Anti-DLL3-variant1 (V1) | DLL3_HC_H85N | DLL3_LC |
| 3 | Anti-DLL3-variant2 (V2) | DLL3_HC_H85N | DLL3_LC_E84G |
| 4 | Anti-DLL3-variant3 (V3) | DLL3_HC | DLL3_LC_E84G |

**C**

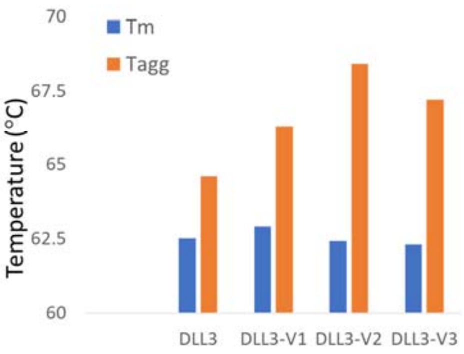


**D**

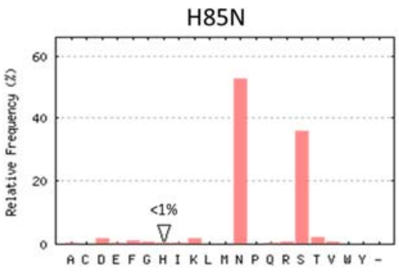


**E**

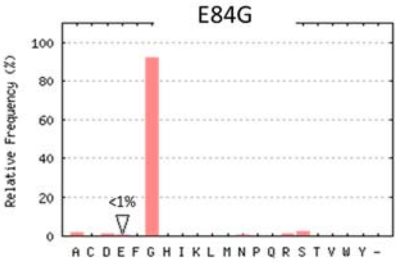

Supplement: Supplementary file 1 — Supplementary Information. [file 41598_2020_66636_MOESM1_ESM.pdf]
